# Prediction of midpalatal suture maturation stage based on transfer learning and enhanced vision transformer

**DOI:** 10.1186/s12911-024-02598-w

**Published:** 2024-08-22

**Authors:** Haomin Tang, Shu Liu, Weijie Tan, Lingling Fu, Ming Yan, Hongchao Feng

**Affiliations:** 1 College of Medicine, Guizhou University, Guiyang, China; 2 Department of Orthodontics, Guiyang Hospital of Stomatology, Guiyang, 550002 China; 3 Guizhou Big Data Academy, Guizhou University, Guiyang, 550025 China; 4 Department of Oral and Maxillofacial Surgery, Guiyang Hospital of Stomatology, Guiyang, 550002 China

**Keywords:** Midpalatal suture maturation stages, Self-attention, Vision transformer, Cone beam computed tomography images

## Abstract

**Background:**

Maxillary expansion is an important treatment method for maxillary transverse hypoplasia. Different methods of maxillary expansion should be carried out depending on the midpalatal suture maturation levels, and the diagnosis was validated by palatal plane cone beam computed tomography (CBCT) images by orthodontists, while such a method suffered from low efficiency and strong subjectivity. This study develops and evaluates an enhanced vision transformer (ViT) to automatically classify CBCT images of midpalatal sutures with different maturation stages.

**Methods:**

In recent years, the use of convolutional neural network (CNN) to classify images of midpalatal suture with different maturation stages has brought positive significance to the decision of the clinical maxillary expansion method. However, CNN cannot adequately learn the long-distance dependencies between images and features, which are also required for global recognition of midpalatal suture CBCT images. The Self-Attention of ViT has the function of capturing the relationship between long-distance pixels of the image. However, it lacks the inductive bias of CNN and needs more data training. To solve this problem, a CNN-enhanced ViT model based on transfer learning is proposed to classify midpalatal suture CBCT images. In this study, 2518 CBCT images of the palate plane are collected, and the images are divided into 1259 images as the training set, 506 images as the verification set, and 753 images as the test set. After the training set image preprocessing, the CNN-enhanced ViT model is trained and adjusted, and the generalization ability of the model is tested on the test set.

**Results:**

The classification accuracy of our proposed ViT model is 95.75%, and its Macro-averaging Area under the receiver operating characteristic Curve (AUC) and Micro-averaging AUC are 97.89% and 98.36% respectively on our data test set. The classification accuracy of the best performing CNN model EfficientnetV2_S was 93.76% on our data test set. The classification accuracy of the clinician is 89.10% on our data test set.

**Conclusions:**

The experimental results show that this method can effectively complete CBCT images classification of midpalatal suture maturation stages, and the performance is better than a clinician. Therefore, the model can provide a valuable reference for orthodontists and assist them in making correct a diagnosis.

## Background

Transverse maxillary hypoplasia is a common problem in orthodontic clinical practice [1]. Clinical manifestations of transverse maxillary hypoplasia include a smaller upper dental arch than a lower dental arch, a higher arch of the palate cap, and crowded dentition, negatively affecting the aesthetic appearance and oral function of patients. Maxillary stenosis is often accompanied by a narrow nasal respiratory tract, which may lead to sleep apnea syndrome [2, 3] and affect patients’ normal lives. The most effective method to treat transverse maxillary hypoplasia is maxillary expansion [4], which means that the maxilla widens to match the width of the upper and lower jaws by opening the midpalatal suture (MPS) with external force, and the maturation of the MPS directly [5, 6] affects the selection of the treatment method of maxillary expansion.

The maturation of MPS is a process of gradual calcification and fusion of the maxillary suture. It is generally believed that patients before the peak of adolescent development can be treated by traditional rapid maxillary expansion RME [7–9], because the MPS has not yet calcified and closed. However, after the peak of adolescent development, the MPS is fused and calcified [10], and the resistance of maxillary expansion also increases thereafter. If the conventional RME is still used for maxillary expansion, it will not only greatly increase the difficulty of maxillary expansion, but also be prone to adverse effects such as tooth inclination, gum atrophy, and tooth root absorption. Therefore, surgically assisted maxillary expansion (SARME) [11–13] and miniscrew assisted rapid palatal expansion (MARPE) [12–14] are needed. Due to the characteristics of growth and development that vary from person to person, there are still a small number of people with slow or premature MPS maturation, and there is great uncertainty in judging MPS maturation based on age alone [15]. Therefore, an objective diagnostic system is needed to accurately obtain information about the degree of MPS calcification, to select the most appropriate and effective treatment for patients.

Angelierid et al. [16] proposed a personalized MPS maturation staging method based on CBCT, which divides the development of MPS into five stages: A, B, C, D, and E. Stage C can be used as the demarcation line, and the MPS in stage A, B and C has not fully calcified and closed, so patients in these stages can use traditional RME to open the MPS and expand the maxillary dental arch. It is unnecessary to use SARME or MARPE to expand the arch, to avoid unnecessary damage and economic burden. However, for patients in the D and E stages, the MPS has calcified and closed, so it hinders the dilatation force of traditional RME. SARME or MARPE are better choices to obtain more skeletal effects [12, 17].

Although there is a clear staging standard for judging the maturation of the MPS according to the morphology of the MPS in CBCT images, in actual clinical diagnosis, if only one doctor’s judgment is relied on, sometimes the before and after staging results of the same image are different, it will bring certain uncertainty to the final diagnosis. Therefore, this study proposed to rely on deep learning algorithms to improve the accuracy of clinician’s MPS maturation staging.

With the development of artificial intelligence, deep learning has made rapid progress in the field of medical imaging. In the field of medical image classification, the convolutional neural network (CNN) has the ability of local feature extraction and has achieved high accuracy in many previous CBCT image classification tasks [18–23]. However, the local characteristics of the convolutional layer limit the network to capture global information, and the theoretical receptive field of deep pixels [24] can cover the whole image, but in reality, the truly effective receptive field is much smaller and also increases the computational cost of CNN. At the same time, previous study [25] have shown that compared with Transformer, CNN has weaker long-range dependence. Therefore, Compared with Transformer, CNN may not fully capture the the long-distance dependencies among these features (global information) of the MPS in CBCT image. In 2020, Dosovitskiy et al. [26] proposed vision transformer (ViT) architecture for image classification applications for the first time to solve the above defects of CNN. This architecture takes advantage of the Transformer’s long sequence modeling capability and global attention mechanism. Thus, the global feature representation is obtained. However, the ViT lacks the inductive bias that CNN has [27], so it cannot be well generalized on small data sets, and there is a risk of overfitting, and it happens that it is difficult to obtain data sets in the field of medical images. To make up for the lack of data sets, two technologies of data augmentation [28] and transfer learning [29] are used in this study. Data augmentation is divided into offline augmentation and online augmentation. Offline augmentation mainly increases the number of training samples, while online augmentation mainly increases the types of training samples. Both types of data augmentation generate new training samples from existing training samples. Transfer learning is a method that allows the model to be pre-trained on a sufficiently large data set, and the pre-trained weights are loaded onto the untrained model and then trained on the target data set. This method transfers the knowledge or experience learned from one task to another task. Since transfer learning does not require the same distribution of pre-trained data and target task data, most CBCT image classification tasks based on deep learning adopt transfer learning to solve the problem of insufficient training of new tasks.

Based on the above analysis of the advantages and disadvantages of CNN and ViT, both traditional convolution and Transformer have their advantages and disadvantages. At present, a large number of successful models of CNN combined with Transformer have been proposed in many new studies to make use of their advantages to alleviate their limitations. Some models use CNN to extract image features and then use a Transformer for classification. This approach takes advantage of CNN’s ability to efficiently process spatial structure data and Transformer’s strong advantage for sequence modeling. For example, the Spatially Adaptive Transformer proposed by Long Sun et al. [30] uses CNN to extract features and improves the Transformer’s ability to process local information through the spatial attention mechanism. It can adaptively weight input features in different locations to process the local information of the image. In addition, ConViT proposed by Stéphane d’Ascoli et al. [31], and IEViT proposed by Gabriel Iluebe Okolo et al. [32]. and CoAtNet proposed by Zihang Dai et al. [33] all used similar methods. In this study, we also adopt a CNN-enhanced ViT method to improve and use the ViT original variant ViT_B/16 as the backbone network model, and introduce multi-scale overlapping sliding convolution kernel for local feature extraction, which also adds more inductive bias to the network to improve the generalization ability of the model.

In this study, the CBCT image data set of MPS we collected was unbalanced. This causes the model to be more sensitive to the excessive number of category samples and perform poorly on the small number of category samples. To this end, we use a data set unbalanced sampler (torchsampler [34]) to balance the number of samples in each category, to improve the model’s poor performance on a small number of categories. In this work, this paper has three main contributions as follows:

1. Publicly available data on CBCT images in the field of stomatology are very scarce. In this paper, CBCT screenshots of the MPS were collected and image screening and preprocessing were carried out. At the same time, a torchsampler is used for balanced sampling of our CBCT image dataset, which overcomes the bias of model learning caused by class imbalance. Moreover, this method is superior to random oversampling and undersampling.
2. The current literature mainly adopts the CNN-based CBCT image classification model, but CNN will miss the global feature information of images. To make up for this shortcoming, we propose a vision transformer based on transfer learning, which has the ability of global feature learning and shows good recognition accuracy.
3. This paper uses CNN to enhance the ViT_B/16 model: multi-scale overlapping sliding convolutional check images are added for multi-scale local feature extraction, and local information is integrated into the network to enhance induction bias.

## Materials and methods

### Dataset

**Fig. 1:**
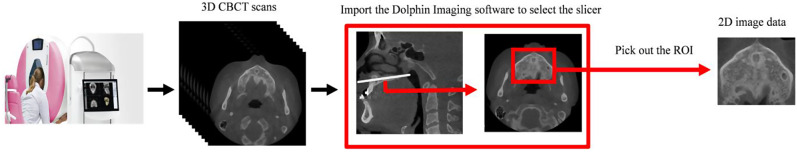
Our data acquisition process

In this study, CBCT images were selected from the case data of the Orthodontics Department of Guiyang Stomatological Hospital from January 2021 to July 2023, and the three-dimensional DICOM (Digital Imaging and Communications in Medicine) data obtained after scanning by CBCT machine were imported into Dolphin imaging 11.9 software of the computer to extract the slicer of the MPS area. And then three orthodontists then manually captured the region of interest (ROI) from this slice to make the dataset. The specific process is shown in Fig. 1.

**Fig. 2:**
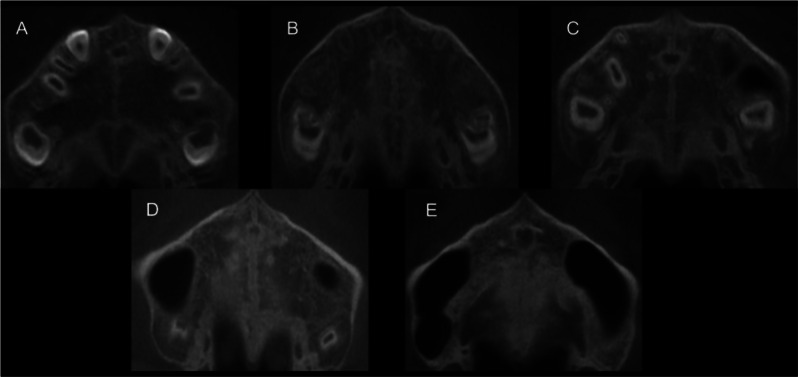
(**A**) In stage A, the MPS presented a relatively straight, high-density white line with no or little curvature; (**B**) In stage B, there is a serrated white line of high-density in the MPS, and there may be two parallel high-density lines or low-density masses locally; (**C**) In stage C, two parallel serrated white lines of high-density appear in the midpalate suture, separated in some areas by small low-density masses; (**D**) In stage D, the MPS located in the posterior palatine bone has a similar density to the surrounding bone and is no longer visible. The MPS in the maxillary part has not yet fused and still presents two serrated high-density lines; (**E**) In stage E, the MPS of the maxilla is no longer visible, and the density is consistent with the surrounding bone

According to the maturation staging method proposed by Angelieri et al. [16], a screenshot was taken of the MPS area (The screenshot is the palatal plane passing through the posterior nasal spine), and clear CBCT images of five different maturational stages were obtained, as shown in Fig. 2.

**Fig. 3:**
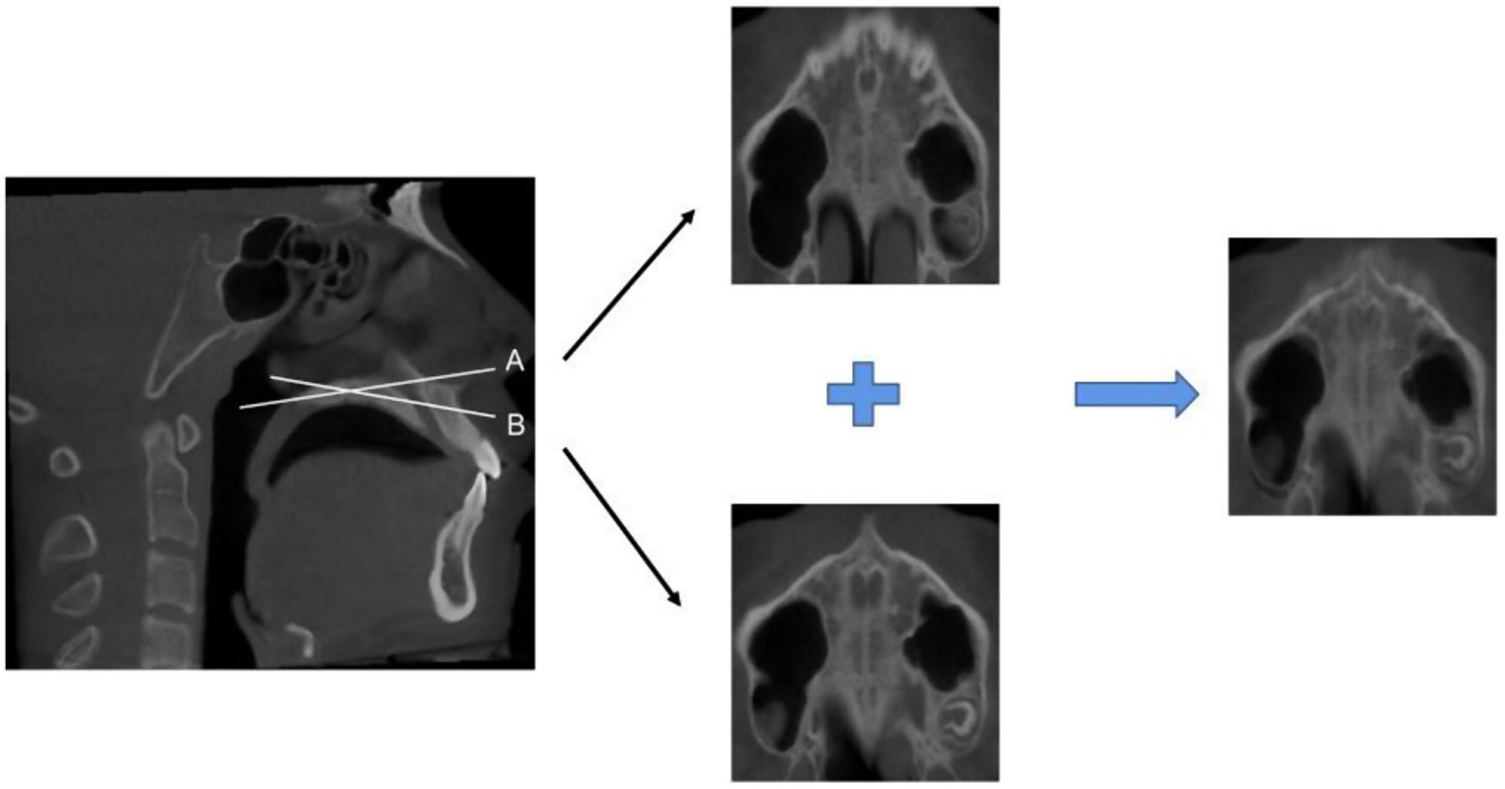
Image fusion of curved palate plane

In our dataset, the number of CBCTs intercepted on the curved palate plane were 22 and only accounted for a small proportion. For patients with curved palate plane, we need to take screenshots of the front and back positions of the palate plane at the same time, and then use a simple and efficient weighted average method to perform image fusion [35] (Fig. 3), which fuses the information contained in these two pictures into one image and helps to show the overall structure of the MPS.

The CBCT images of MPS collected in this study are staged and labeled by 3 orthodontists. For the images with inconsistent staging results among the 3 orthodontists, a consensus is reached after a joint discussion. Finally, the dataset of CBCT images is divided into five different stages (A, B, C, D, and E), a total of 2518 CBCT images of MPS are collected in the dataset, including 416 in A, 168 in B, 514 in C, 598 in D and 822 in E. Among the 2518 images, the training set, verification set, and test set are divided according to the ratio of 5:2:3. 1259 images are used for training, 506 images are used for verification, and the remaining 753 images are used for testing, as shown in Table 1.

**Table 1:** The partition of the MPS CBCT images dataset

	A	B	C	D	E	Total
Train	208	84	257	299	411	1259
Valid	84	34	103	120	165	506
Test	124	50	154	179	246	753

### Data preprocessing

#### Data augmentation

In deep learning image classification, the larger scale and the higher quality of the application data it is, the better generalization ability and classification performance the model has. The data directly determines the upper limit of the model learning. There are two specific ways to use data augmentation [28]. One is to perform all transformations in advance and essentially increase the size of the data set. This method is called offline augmentation, which is more suitable for smaller data sets and will eventually increase the amount of data by a certain multiple. The other is a mini-batch transformation as data is fed into the model, which is called online augmentation and is more suitable for large data sets. In this study, to overcome the problems of relatively small data and overfitting of the model, we first augment the training set offline, and then augment it online. Offline augmentation includes operations such as Gamma enhancement, median filtering, and flipping. Online augmentation includes operations such as cropping, rotation, and brightness modulation. The specific data augmentation methods are shown in Table 2.

**Table 2:** Data augmentation details

Data augmentation method	Offline/Online augmentation	Operation
Horizontally flipping	offline	Double the number of images
Vertically flipping	offline	Double the number of images
Median filtering	online	Double the number of images
Gamma enhancement	offline	Double the number of images
Random shift in brightness	online	Adjust the image brightness in a range of 0.8 to 1.2
Random rotating	online	Rotate the image from − 15° to 15° with a probability of 0.2
Random clipping	online	Crop the image with an aspect ratio of 0.9

#### Data normalization

Data normalization [36] is the process of scaling data proportionally to fit into a small specific range. Its purpose is to avoid numerical issues caused by large numbers. When we use the gradient descent method to solve optimization problems, the normalization can accelerate the solving speed of gradient descent to improve the convergence speed of the model. This research is a classification algorithm task, which requires distance to measure similarity, z-score normalization performs better. Therefore, z-score normalization is adopted in this study. After z-score normalization, the data is converted into a distribution with a mean of 0 and a standard deviation of 1. Its transformation formula (1) is shown below:

1 $$x{\prime } = x - a / b$$

*x* is the value of input data, *x’* is the value of output data, *a* is the mean value of the input data, and *b* is the variance value of the input data.

#### Balanced sampler

In our training data set, the quantitative ratio of five stages (A, B, C, D, E) is 2.5:1:3:3.5:4.9, and the number of stage B is 20.4% of the number of stage E, which is close to a moderate imbalance. The quantitative imbalance between classes can lead to bias in CBCT image classification results. To alleviate this problem, we used balanced sampling to achieve data balance in this study.

One widely used technique is called random resampling. It involves removing individual samples from a majority of samples (random undersampling) and adding more samples to a minority of samples (random oversampling). Although the above two methods can maintain a balanced number of sample classes, they have their disadvantages. The way to achieve random oversampling is to replicate samples of a small number of classes repeatedly, but that can lead to overfitting of the model [37]. At the same time, the way to realize random downsampling is to delete a large number of certain samples, but that can lead to the loss of training process information.

In this study, a torchsampler [34] was used for balanced sampling to make up for the disadvantage of appeal random resampling. The torchsampler has the following advantages: (1) It can rebalance the distribution between classes when sampling from a unbalanced dataset. (2) It can automatically estimate the sampling weights. (3) It can avoid creating a new balanced dataset. (4) Mitigate overfitting when it is used in conjunction with data augmentation techniques.

### Traditional vision transformer (ViT)

Transformer has already achieved considerable success in the field of Natural Language Processing (NLP). Vision Transformer (ViT) applies Transformer’s ideas and architecture in the field of NLP to image classification tasks. When trained on large data sets, its image classification performance is comparable to or even better than CNN architecture. Dosovitskiy et al. [26] used the ViT network to first divide a picture into several small (16 × 16) patches, and then flattened them from 16 × 16 to 1 × 256 respectively. After a Linear Projection, Convert each patch into a patch embedding. Self-Attention calculation is performed in the encoder to enable patches to perform pairwise information interaction, and after several repeated stacking Self-Attention calculations, finally, classification is performed through the Multi-layer Perceptron (MLP) head.

### Our proposed improved vision transformer model

Compared with the traditional ViT model of directly divided images into patches, ViT_B/16 (proposed by Dosovitskiy et al.), as an original variant of ViT with the smallest model parameters, has better feature extraction capability relying on convolution with size 16 × 16 and step size 16. However, ViT_B/16 uses a large-scale convolution kernel to extract image information in blocks, and there is no overlap area between each block, which destroys the overall structure of the image, ignores the pixel information between adjacent blocks, and lacks local inductive bias. So when training small data sets, the underlying self-attention mechanism will fail to learn the local information between patches, resulting in a decline in model accuracy.

**Fig. 4:**
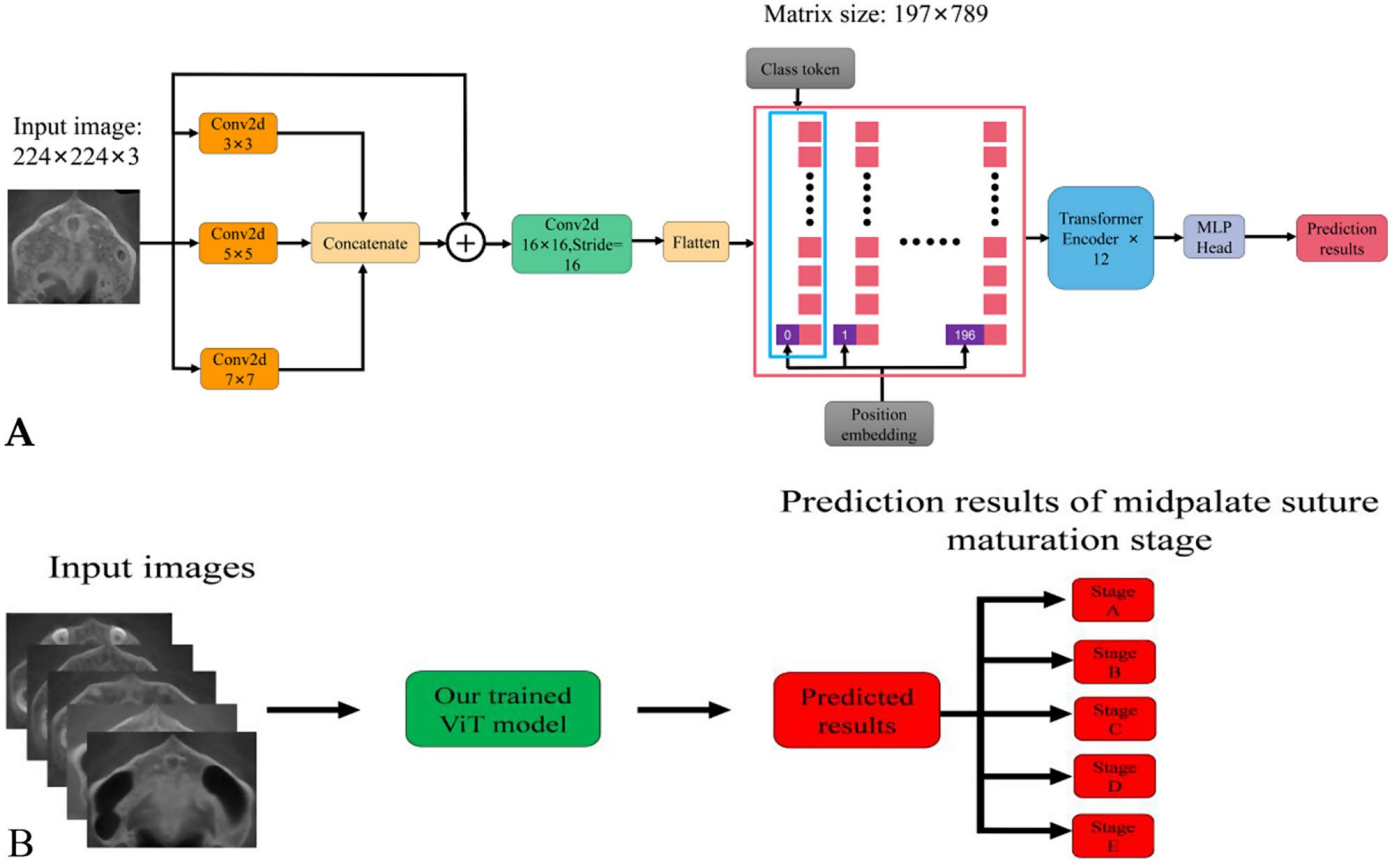
Proposed ViT predicts the maturation stage of MPS: **A** The proposed ViT network structure; **B** Prediction process of MPS CBCT images maturation stage

To alleviate the above problems, this study proposed an improved ViT_B/16 network, as shown in Fig. 4A. We used multiple small-scale overlapping sliding convolutions to extract image information, which could also make up for the insufficient local inductive bias of the network. Since the value of convolution kernel size has no absolute advantages or disadvantages and needs to be designed according to specific application scenarios, we propose a multi-scale convolutional extraction block, which stacks three parallel 2D convolution cores with different convolution kernel sizes, and it is similar to the inception module [38], as shown in Fig. 4A. The kernel size of the first convolution kernel is 3, the step is 1, the padding size is 1, and using 1 filter for dimensionality reduction; The kernel size of the second convolution kernel is 5, the step is 1, the padding size is 2, and using 1 filter for dimensionality reduction; The kernel size of the third convolution kernel is 7, the step is 1, the padding size is 3, 1 filter is used for dimensionality reduction, and the above three convolution are connected with activation function RELU respectively to enhance the nonlinear fitting ability of the network. Then the 224 × 224 × 1 feature map obtained after the convolution of the three convolution kernels is concatenated to the output. Inspired by the ResNet [39] architecture, we introduce a skip connection, which adds the original input to the concatenate fusion and finally forms a residual network.Then, the convolution with convolution kernel size of 16 × 16, step length of 16, and number of convolution kernels of 768 is used to change the image data format from [224, 224, 3] to [14, 14, 768]. Then, fatten is applied to each 14 × 14 patch to flatten the two dimensions of image width and height. The resulting tokens are in the format of [196, 768], and at the same time, a classification vector class token with the format of [1, 768] is spliced in the first dimension. Since all tokens are exchanging information with each other, the vector class token can learn useful information from the other 196 tokens. Finally, the final prediction result can be obtained only by making a final judgment based on the output of the class token. The size of the matrix obtained after concatenation is [197, 768], that is, 197 tokens are obtained, and the dimension of each token vector is 768. In order to obtain the spatial position information of the input image, a learnable position embeding is added to each token, the data format of which does not change, it also is [197, 768], and then input to the stacked Transformer Encoder, and the output is passed to the MLP header for the prediction of the final MPS maturation stage.

Figure 4B shows the prediction process of our proposed trained ViT model. The input of ViT is the ROI images that we manually captured in this study, and these images include five different MSP maturation stages. The automatic output of ViT is the stage (one of the A, B, C, D and E stage) with the highest probability of ViT prediction corresponding to the input CT image.

### Transformer encoder

**Fig. 5:**
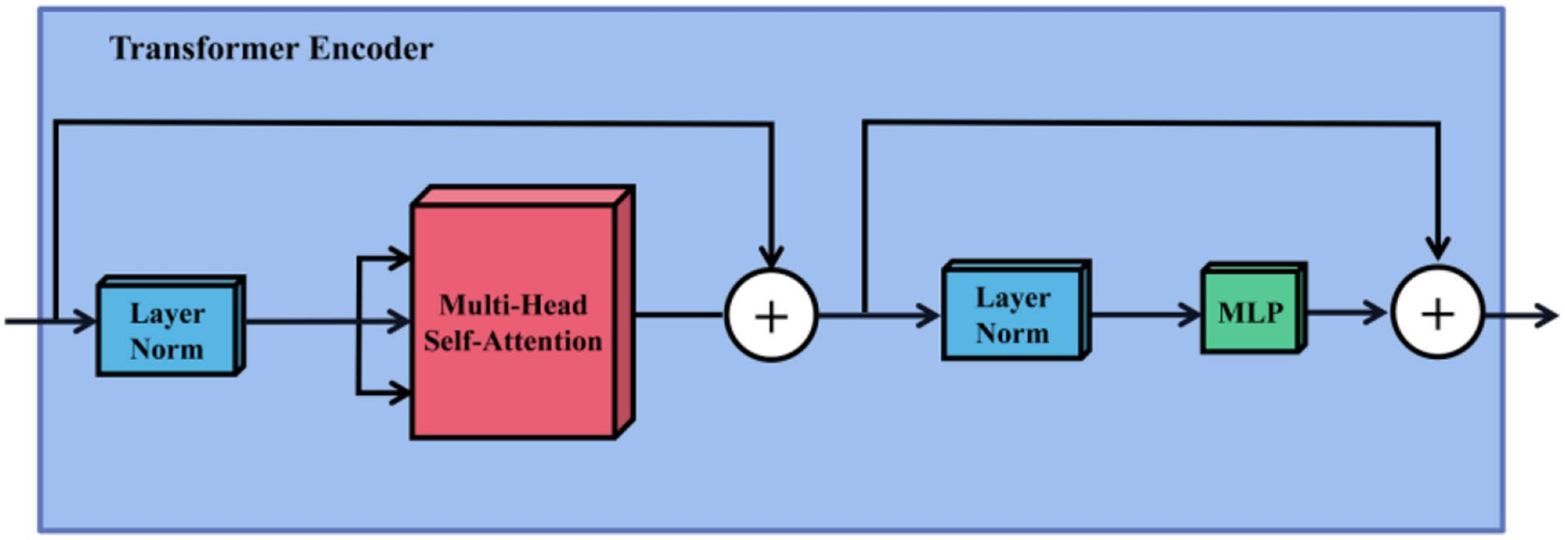
The architecture of the transformer encoder

In this study, the Transformer Encoder stacks Encoder blocks 12 times. It mainly consists of Layer Norm, Multi-Head Self-Attention (MSA) [40], and Multi-layer Perceptron (MLP) Block. Its structure diagram is shown in Fig. 5. The Layer Norm is used to normalize the input data, reducing the training time and improving the training stability. After normalization, MSA is given, it is based on Self-Attention, which is a special case of Attention. The formulas of Self-Attention are shown in the following (2) to (5). It can be seen from the formulas that the core of Self-Attention is to calculate the weight of attention by *Q* and *K*, and then apply it to *V* to get the whole weight and output.

2 $${Q = X}{W}^{q}$$

3 $$K = XW^k$$

4 $$V= XW^{v}$$

5 $$\text{Attention}(Q,K,V)=\text{ Softmax}(\frac{{Q}{{K}}^{\text{T}}}{\sqrt{{d}_{k}}}){V}$$

Specifically, in Eqs. (2), (3), and (4), the input matrix X is linearly mapped by matrix multiplication to the attention space matrix *Q* (query matrix), *K* (key matrix), *V*(value matrix), *W*^*q*^, *W*^*k*^, *W*^*v*^ are learnable hyperparameter matrices. In formula (5), √*d*_*k*_ represents the dimension of the key, and 1/√*d*_*k*_ represents the scaling factor, which is used to prevent the product of *Q* and *K* from being too large, resulting in a very small gradient after Softmax operation, which is not conducive to network training. Finally, the result is a weighted sum of similarity and worth based on query and key.

Self-Attention tends to focus too much on its location, So Multi-Head Self-Attention (MSA) is proposed to remedy this deficiency, and the output that can be given to the attention layer contains encoded information in different Spaces. The MSA of this study is to divide each *Q*,* K*, and *V* into 12 branches, perform 12 different attention calculations on *Q*,* K*, and *V* to get multiple different outputs, and then concatenate these different outputs together to get the final output. The formula is shown in Eq. (6) and Eq. (7).

6 $$\text{head}_{\text{i}\text{ }}\text{= Attention}\left({Q}{{W}}_{{i}}^{{Q}}{,K}{{W}}_{{i}}^{{K}}{,V}{{W}}_{{i}}^{{V}}\right)\text{ }{i}\text{=1,2}^\text{...}\text{,12}$$

7 $$\text{MSA}\left({Q,K}{,V}\right)\text{ = }\text{Concatnate}\left(\text{hea}{\text{d}}_{\text{1}}\text{,....,}\text{hea}{\text{d}}_{\text{12}}\right){{W}}^{\text{O}}$$

**Fig. 6:**
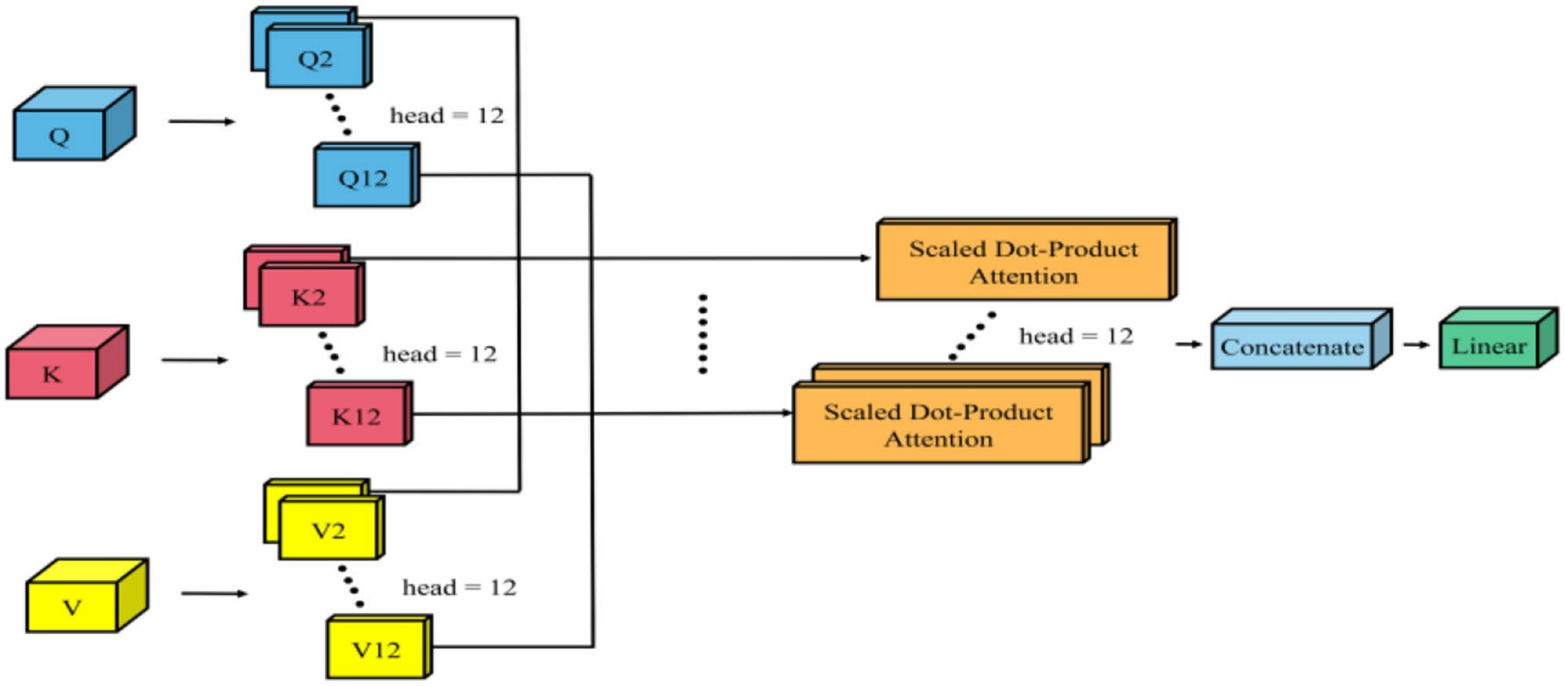
The architecture of the multi-head self-attention layer

In Eq. (6), *W*_*i*_^*Q*^, *W*_*i*_^*K*^, and *W*_*i*_^*V*^ are hyperparameter matrices that can be learned. In Eq. (7), *W*^*0*^, through linear mapping, carries out a weighted fusion of multiple features while ensuring the constant length of the input and output vectors of MSA. Its structure is shown on the left side in Fig. 6.

The MLP Block consists of a fully connected layer, Gaussian Error Linear Unit (GELU) activation function, and Dropout. MLP output data format is the same as the input, both are [197, 768]. MSA and MLP are the main structures of the Transformer Encoder and can be represented by formulas (8) and (9) containing these two structures.

8 $$x_{i}^{\prime }= MSA{\left({LN}{\left({\text{x}}_{\text{i-1}}\right)}\right) }+ x_{i-1}\,i=1,2^{...},12$$

9 $${{x}}_{{i}\text{ }}\text{= }{MLP}\left({LN}\left({{x}}_{{i}}^{{\prime }}\right)\right){+}{{ x}}_{{i}}^{{\prime }}\text{ }\text{ }{i}{ = 1,2}^{...}{,12}$$

In the two formulas, *LN* represents Layer Norm, *x*_*i*_*’* is the result of multi-head self-attention, and *x*_*i*_ represents the output of the Transformer Block each time. In this study, we take *x*_*i*_^*0*^ as the first token of the sequence output of the Transformer Encoder, and *x*_*i*_^*0*^ passes through *LN* to obtain input *F* of the classifier, as shown in Eq. (10), and the classifier is the *MLP* head in the network.

10 $${F}{ }{= }{LN}\left({{x}}_{{i}}^{{0}}\right)$$

### Experimental setup

**Fig. 7:**
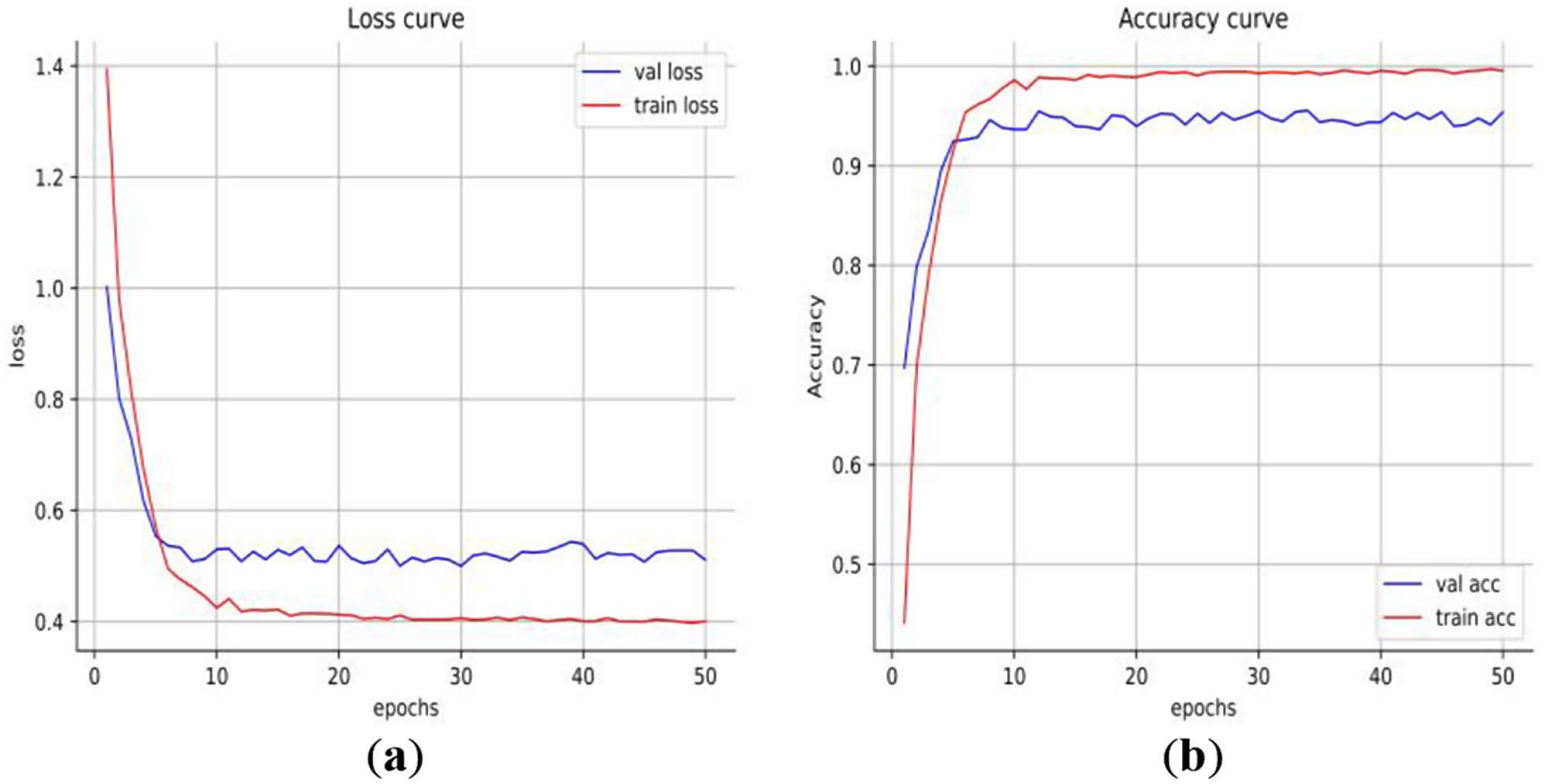
Training/validation loss and accuracy of the proposed model: (**a**) Loss curve; and (**b**) Accuracy curve

Based on the Pytorch environment, the graphics card used in this experiment is NVIDIA RTX 3090, the model optimizer is SGD, the learning rate is set to 0.001, and the learning momentum is 0.9. A batch size is 16. All image specifications for the training input are adjusted to 224 × 224 × 3, and the training is finished after 50 epochs. By observing the change curves of accuracy and loss value on the training set and verification set in the training, we can not only judge whether the model is underfitting or overfitting but also realize the modulation of hyperparameters and obtain the optimal model hyperparameter setting(Learning rate, a batch size, etc.). Figure 7 shows the loss value change curve and accuracy change curve of our proposed ViT model on the training set and verification set under the modulation optimal hyperparameter setting.

### Training and classification

To evaluate the performance of our proposed ViT model, we compared our proposed model with ViT_B/16, B/32, L/16, L/32(Dosovitskiy et al. proposed), and other ViT variants. Transfer learning is used for all models and the specific implementation methods are as follows: All models are parameter initialized using pre-trained weights on the ImageNet [41] dataset (except the models’ output layer), and we reset the models’ output layer output number (the number of classes of our data), then the parameters of the models output layer and additional network layers are randomly initialized. Finally, our data set and the pre-trained model’s data set (ImageNet) distribution are not quite consistent, so we fine-tune the parameters of all layers of the model to train.

In this study, cross-entropy is used as the training loss function (11). Cross-entropy loss is mainly used to measure the difference information between two probability distributions, that is the gap between model prediction and label. The smaller the gap is, the better. Therefore, cross entropy can be used as a loss function. In the formula (11), C represents the class number of training samples (there are five different stages in this study: A, B, C, D, and E), and P represents the prediction probability value. Where P=[P_0_,…, P_C-1_] is a probability distribution, and each element *Pi* represents the predicted probability value of the sample belonging to class *i*, which is obtained by Softmax. *Y* represents the label of the training sample, where *Y*=[Y_0_,…,Y_C-1_] is the one-hot representation of the sample label, Yi = 1 when the sample belongs to class i, otherwise Yi = 0; *Pc* is the probability that the prediction is correct.

11 $$\text{ }{Loss}\text{ =}-\sum _{{i}\text{=0}}^{\text{C-1}}{{Y}}_{\text{i}}\text{log}\text{(}{{P}}_{{i}}\text{)}\text{=}-\text{ log}\text{(}{{P}}_{{c}}\text{)}$$

To further mitigate the overfitting [37] of deep learning models. In this study, we adopt the label smoothing [42] proposed by Christian Szegedy et al. in 2016. Label smoothing is regarded as a regularization technique, which limits overconfidence in model predictions to improve the generalization ability. Currently, this method is adopted by many research deep learning classification tasks. After adding label smoothing, the loss function becomes the formula (12), e is a small hyperparameter, here we take 0.1, *Pi* is the probability of predicting the correct class, *Pj* is the probability of predicting the other class, and N is the number of other classes except the correct class.

12 $${Label}\text{ }{Smoothing }\,{Loss}\text{ }\text{= }-\text{[}\left(\text{1} - {e}\right)\text{log}\left({{P}}_{{i}}\right)\text{+ e}\sum _{{j}\text{=1}}^{\text{N}}\frac{\text{log}\left({{P}}_{{j}}\right)}{\text{N}}\text{]}$$

### Evaluation metrics

To verify the effectiveness of the improved model for classifying MPS CBCT Images, We used six different metrics to evaluate the model: Accuracy, Precision, Recall(Sensitivity), Specificity, F1-score, the area under the receiver operating curve(AUC) [43]. (13) to (17) are formulas for calculating these six metrics.

13 $$\text{Accuracy = }\frac{{TP + TN}}{{TP + FN + TN + FP}}$$

14 $$\text{Precision = }\frac{{TP}}{{TP}{ + FP}}$$

15 $$\text{Recall(Sensitivity) = }\frac{{TP}}{{TP}{ + FN}}$$

16 $$\text{S}\text{pecificity }\text{= }\frac{{TN}}{{FP + TN}}$$

17 $$\text{F1-score = 2}{\cdot}\frac{\text{ Precision}{\cdot}\text{Recall}}{\text{Precision }\text{+ Recall}}\left(17\right)$$

**Fig. 8:**
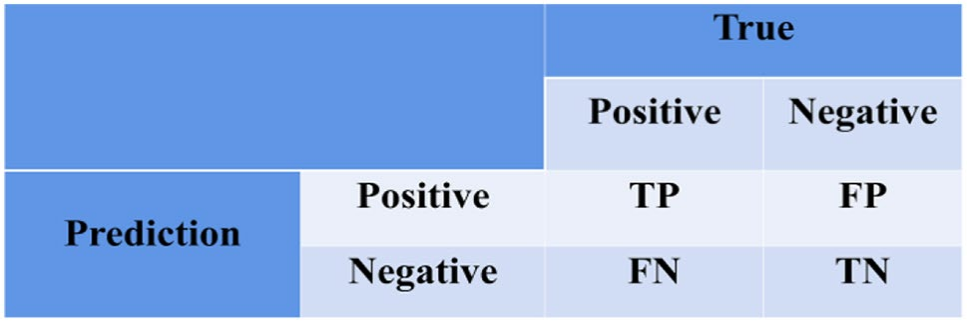
The confusion matrix of indicators

*TP* (True Positive) refers to the number of positive samples that are correctly classified; *TN* (True Negative) refers to the number of negative samples correctly classified; *FP* (False Positive) refers to the number of Negative samples that are incorrectly classified, *FN* (False Negative) refers to the number of positive samples that are incorrectly classified. The confusion matrix is shown in Fig. 8.

In addition, AUC represents the area under the Receiver Operating Characteristic Curve (ROC), and its value ranges from 0 to 1. The classification effect of the model can be evaluated according to the range of AUC values.

## Results

### Result of not using and using torchsampler

To verify the validity of the torchsampler [28] we used in this study, we take our data set as an example to illustrate the specific performance of the sampler above.

**Fig. 9:**
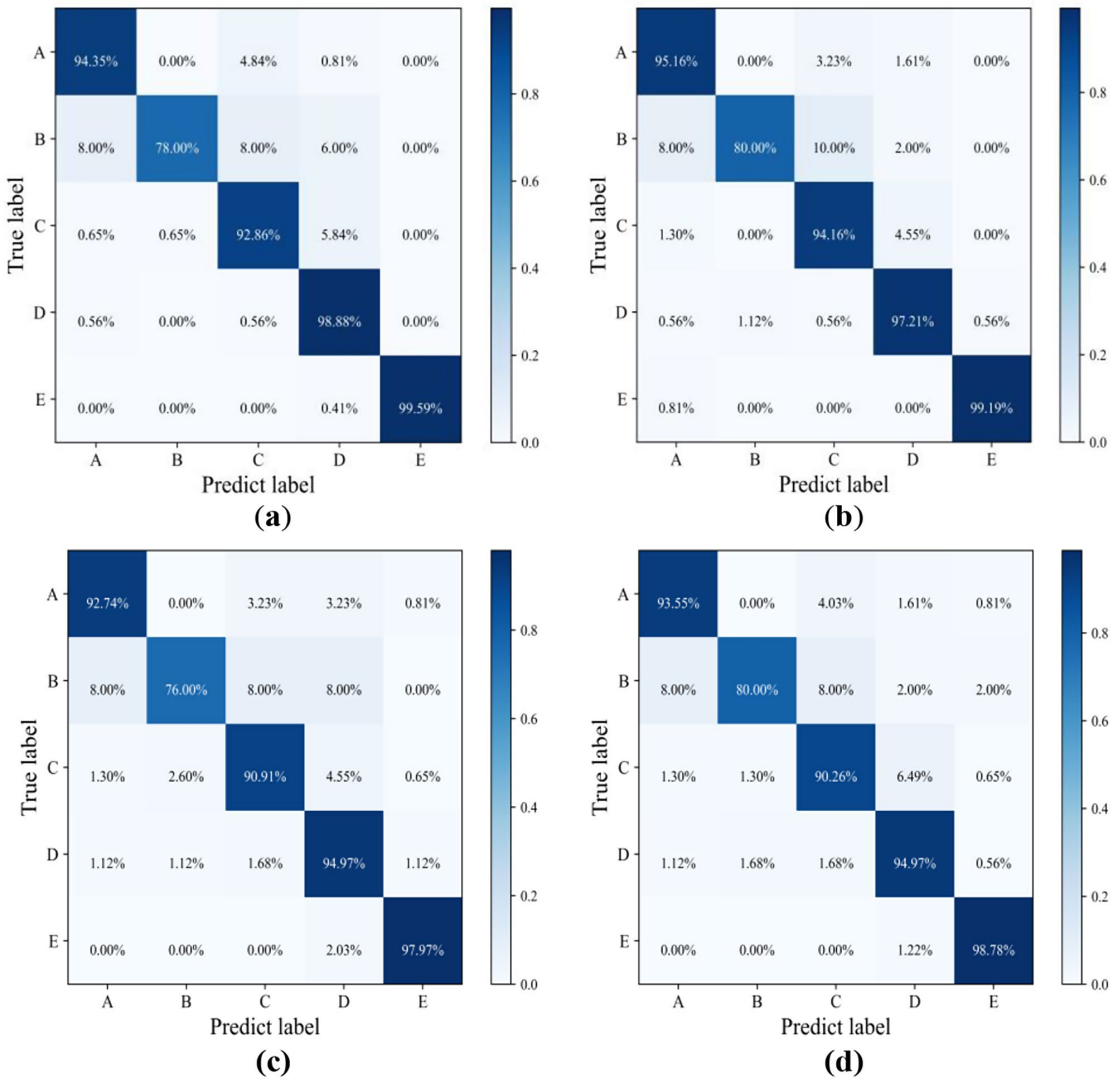
Confusion matrices of test results with and without balanced sampling on our dataset: (**a**) No balanced sampling; (**b**) Torchsampler balanced sampling; (**c**) Random undersampling; (**d**) Random oversampling

In this study, the ViT model proposed by us was trained under the original unbalanced data set, trained after random undersampling of the original data set, trained after random undersampling of the original data set, trained after using torchsampler balanced sampling the original data set, and tested on the test set after the training. Four test data confusion matrices with random undersampling, random oversampling, torchsampler balanced sampling and no balanced sampling were obtained respectively, as shown in Fig. 9.

In Fig. 9, we can find that the recognition accuracy is more balanced in the five stages after the use of torchsampler: the main diagonal color distribution of the confusion matrix in Fig. 9(b) is more uniform than that in Fig. 9.

(a). After the balanced sampling of torchsampler is used, the accuracys of the stage A, B and C are significantly improved, and the accuracy of the stage B with the smallest number of samples is increased by the highest 2%. Although the accuracy of other stages (D, E) with a large number of samples is decreased, the overall accuracy remains unchanged. In Fig. 9, we can also find that the effect of using torchsampler in this data set is better than that of using oversampling and undersampling: the accuracy of each stage on the confusion matrix of Fig. 9(c) and (d) is less than or equal to that of Fig. 9(b). Figure 9 shows the effect of using oversampling is better than that of using undersampling: the accuracy of each stage on the confusion matrix in Fig. 9(d) is greater than that in Fig. 9(c).

### Comparison of the proposed vit with other vit variants

In this study, the classification performance of our proposed ViT model, four original ViT model variants (ViT_B/16, B/32, L/16, L/32), and two other ViT model variants (Swin Transformer proposed by Ze Liu et al. [44], and Convit proposed by Stéphane d’Ascoli [31] et al.) was evaluated using the following metrics: accuracy, recall (sensitivity), accuracy, F1-score. Among them, F1-score, recall, and precision are calculated for each class, and then the arithmetic average is calculated for all classes, and the average (macro average) is used as the final metric. Table 3 shows the performance results of the seven models proposed based on transfer learning on our test set (MPS CBCT images). Among the four variants of the original ViT model, ViT_B/16 has the best evaluation metric, so the ViT proposed in this study is also improved based on ViT_B/16(CNN-enhanced ViT_B/16). To prove the validity of the classification performance of our proposed ViT model, we not only compare our proposed ViT model with the original ViT_B/16 but also compare it with Swin-base and Convit-base, which have little difference in the number of model parameters with our proposed ViT. The number of model parameters refers to the total number of training parameters required in model training, which is used to measure the size of the model. The unit M is used here as the measurement. In Table 3, we can find that the test accuracy of our proposed model is 0.9% higher than that of ViT_B/16, 0.4% higher than that of Swin-base, and 0.67% higher than that of Convit-base respectively.

Floating point operations per second (FLOPs) is used to measure the overall complexity of our proposed ViT (the amount of computation) and is often used as an indirect measure of the speed of a deep learning model. The unit is calculated in M. Table 3 shows that compared with ViT_B/16, while the accuracy of our proposed model is improved and the size of model parameters is hardly changed, FLOPs only increase by 11.7 M.

**Table 3:** Classification performance of ViT models on our test set(%)

Model	Accuracy	Precision	Recall	F1-score	Params(M)	FLOPs (M)
ViT_B/16	94.85	94.5	92.96	93.73	86.42	16863.63
ViT_B/32	92.03	92.04	88.68	90.54	88.19	4366.95
ViT_L/16	93.62	92.98	91.26	92.29	304.12	59686.71
ViT_L/32	93.22	93.10	90.77	91.91	327.85	15286.04
**Proposed ViT Model**	**95.75**	**95.15**	**93.15**	**94.13**	**86.42**	**16875.33**
Swin-base	95.35	94.89	92.56	93.71	87.7	15169.77
Convit-base	95.08	94.11	91.48	92.77	86.42	16796.16

### Comparison of the proposed vit with CNN models

To further evaluate the performance of our proposed model, we propose eight traditional CNN models as comparison models. There of these CNN models are Vgg16 [45, 46], EfficientNetB4 [47], MobilenetV2 [48]. The other five CNN models from previous study [23] are ResNet18, ResNet50, ResNet101, InceptionV3, EfficientnetV2_S. After data preprocessing in this study, the four CNN models are trained by the same transfer learning method as the proposed ViT.

Table 4 lists the accuracy, accuracy, recall, and f1-score of all models on our test set. Our proposed ViT accuracy is 95.15%, accuracy is 95.75%, recall rate is 93.15%, and f1 score is 93.15%. We can see that the proposed ViT model performs better than the remaining four traditional CNNs. In terms of accuracy, our proposed ViT model was 5.18% higher than MobileNetV2, 2.79% higher than ResNet50, and 2% higher than EfficientNetB4 and 2.93% higher than Vgg16, respectively. Moreover, and the f1 score of the ViT model proposed by us are also the highest in the four CNN models.

**Table 4:** Classification performance of our proposed ViT and CNN models on our test set (%)

Model	Accuracy	Precision	Recall	F1-score
MobileNetV2	90.57	89.16	86.24	87.67
ResNet50	92.96	91.17	90.56	90.86
ResNet18	90.50	88.58	88.66	88.61
ResNet101	93.62	91.64	91.86	91.75
InceptionV3	92.03	90.70	90.80	90.74
EfficientnetV2_S	93.76	92.84	93.05	92.94
EfficientNet_B4	93.75	92.30	91.38	91.83
Vgg16	92.82	92.88	89.46	91.13
**Proposed ViT Model**	**95.75**	**95.15**	**93.15**	**94.13**

**Fig. 10:**
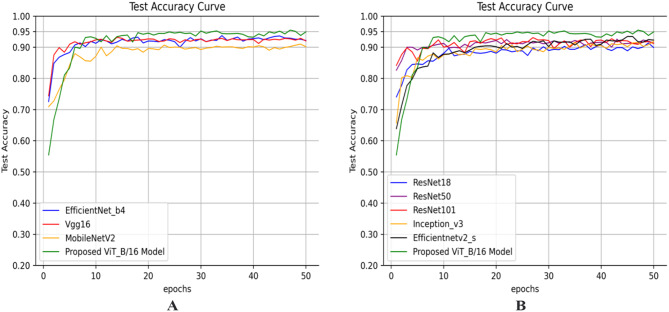
The test accuracy curve of nine models on our test set: **A** Test accuracy curves of MobileNetV2, EfficientNet_b4, Vgg16 and our proposed ViT models; **B** Test accuracy curves of ResNet18, ResNet50, ResNet101, Inceptionv3, Efficientnetv2_s and our proposed ViT model

Figure 10 shows the test accuracy curves in the training process of the nine models. The green curve represents the ViT model proposed in this study, and the other seven curves with different colors represent different traditional CNN models. It can be intuitively found that the accuracy of our proposed ViT model eventually converges the highest. On the whole, the initial accuracy of the CNN models is higher than that of the proposed ViT model, but the convergence speed of the CNN models is faster than that of the proposed ViT model, so the final convergence accuracy of CNN models is lower than that of the proposed ViT model.

**Fig. 11:**
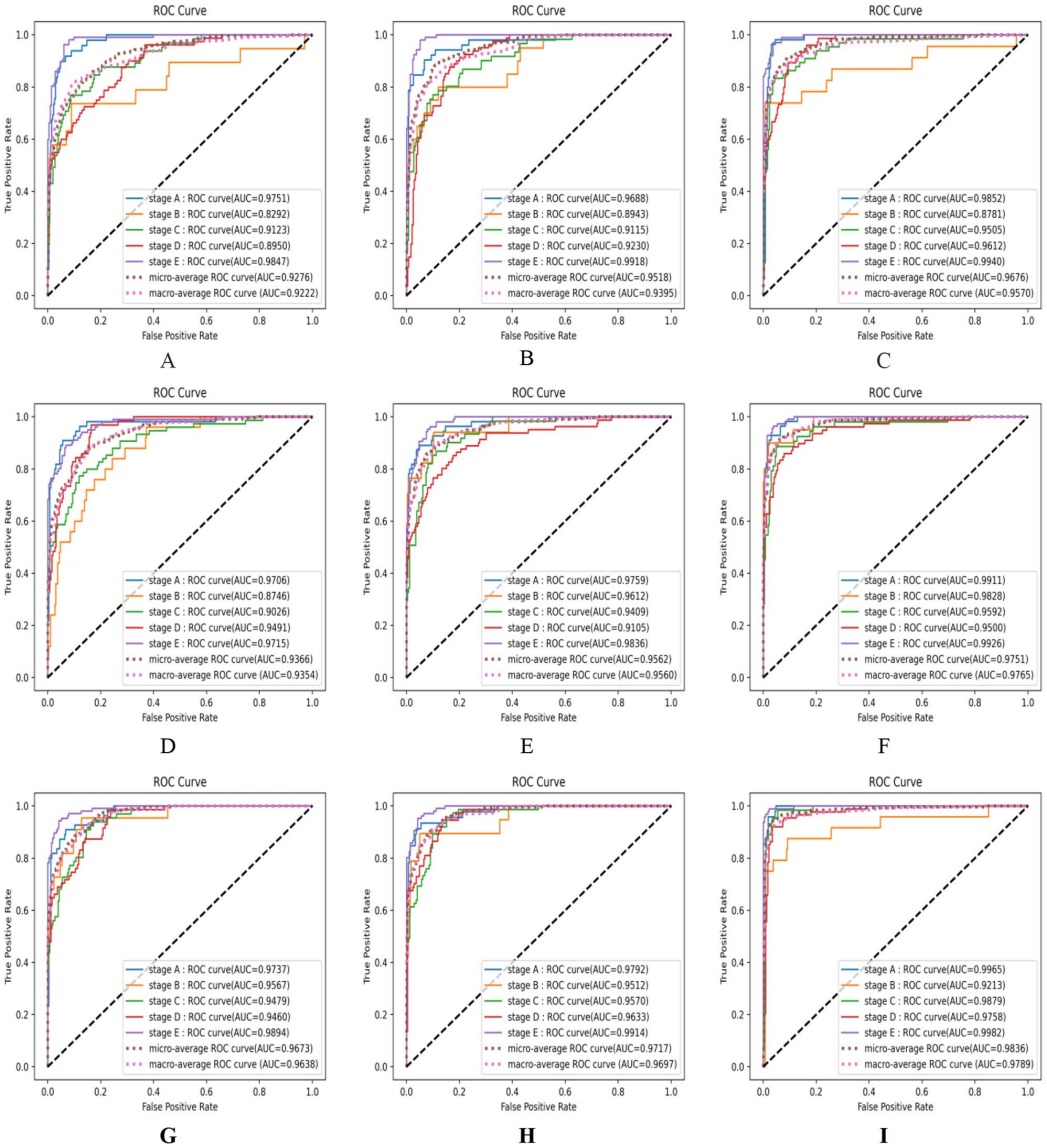
The ROC curves and corresponding AUC values of nine models (**A** MobileNetV2; **B** ResNet50; **C** ResNet101; **D** ResNet18; **E** Inceptionv3, **F** Efficientnetv2_s; **G** EfficientNet_b4; **H** Vgg16; **I** Our proposed ViT model;) on our test set

In addition, Fig. 11 shows the ROC curves and AUC values of the above 8 CNN models and our proposed ViT model. We can find that compared with the eight CNN models, the ViT proposed by us not only had the highest AUC value in the five maturation stages respectively, its Macro-averaging AUC and Micro-averaging AUC also were the highest, which were 97.89% and 98.36%, respectively. EfficientnetV2_S model achieved the best performance among CNNs, its Macro-averaging AUC and Micro-averaging were 97.65% and 97.51%, while MobileNetV2 model had the lowest, which were 92.22% and 92.76%, respectively.

### Grad-cam visualization

**Fig. 12:**
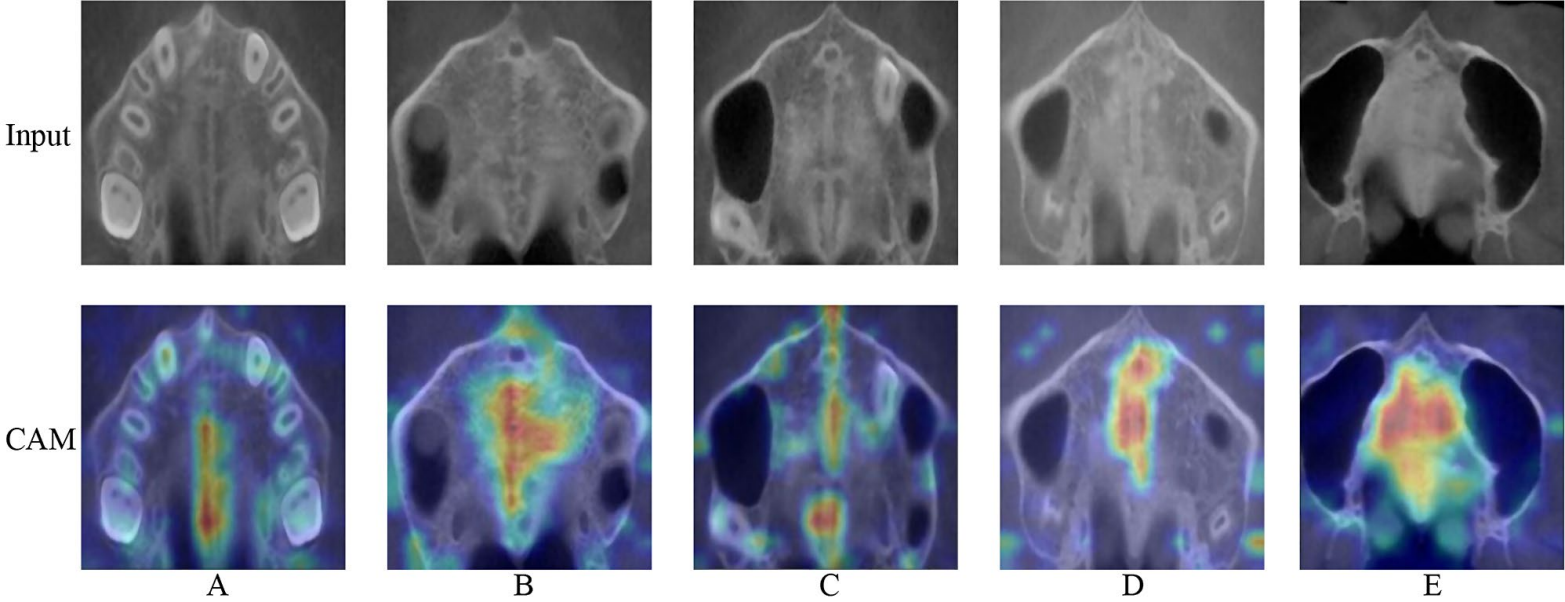
The original input images of the MPS maturation stage **A** to **E** and the corresponding CAM of the input images

The Grad CAM Map-based illustrations proposed by Selvaraju et al. [49] generate “visual explanations” for decisions from large deep learning models. In this study, we use Grad-CAM to make a visual analysis of our proposed ViT. Figure 12 shows the original input images of the MPS maturation stage A to E and the corresponding class activation maps (CAM) of the input images. CAM is a map of the same size as the original image and refers to the thermal map of class activation generated by the input image. It can be understood as the contribution distribution to the predicted output, which can reflect whether the model has learned useful feature information. As shown in Fig. 12, the redder region of Grad-CAM appears in the MPS region. It shows that our proposed model pays more attention to this region, and relies more on the image features of this region in the classification. The effective classification ability of the ViT model proposed in this study can be seen in the Grad-CAM.

## Discussion

In this paper, a CNN-enhanced ViT model is proposed to classify CBCT images of MPS, and the classification effect is good. In this study, CBCT images of five different stages (A, B, C, D, and E) were collected according to the stage method of MPS maturation proposed by Angelieri et al. [16]. Due to the imbalance in the amount of training data of the five categories, we used a torchsampler, which effectively improved the accuracy in a small number of categories. Because balanced sampling avoids the model learning such a priori information about the proportion of training samples, it helps to alleviate the emphasis on most categories in model prediction. In addition, the CBCT data set of midpalatal suture in this study has a small number of images, while the ViT model requires a large amount of data for training to perform well. Moreover, the model trained from scratch in the CBCT data set of midpalatal suture is more likely to lead to overfitting. Therefore, we not only conducted image preprocessing on the original image, but transfer learning was also carried out on the ViT model to avoid that situation.

Nonetheless, the authors of this paper are aware that there are some shortcomings and limitations in this study. The data set used in this study came from only one institution, Guiyang Stomatological Hospital. Highly parametric deep neural networks are highly dependent on the amount of training data available. Performance typically improves as the number of data points increases, and they are more susceptible to the amount of training data available than classical machine learning techniques. This is because of the need to learn useful features as well as decision boundaries, and hence the need for techniques to overcome the lack of data, especially in clinical applications. However, in this study, due to various factors, including the cost of money and time to obtain data, difficulties in cross-site sharing (sharing of focal data between hospitals) and insufficient number of patients, our data set was limited.

This study’s results showed that the clinician’s staging accuracy in the MPS maturation was lower than that of all the deep learning models on the test set. This is because a clinician has strong personal subjectivity when dealing with multi-phase case image recognition, and they will be physically tired of a large number of image recognition, which will lead to more errors. So in this case, deep learning algorithms show advantages. Our proposed ViT in this study combines the advantages of CNN, using overlapping sliding convolution to obtain local image information and serialize it into multi-head self-attention mechanism learning, the model generalization ability is enhanced. Therefore, the classification accuracy of the ViT proposed by us on the MPS CBCT test set is better than that of most CNN and some ViT variants, and can better assist diagnosis in clinical practice.

This study only added CNN block based on the original ViT_B/16 model, so the calculation cost will slightly increase, and the classification accuracy will be improved at the cost of calculation. The author of this paper is aware that the computational cost and speed of CBCT image processing by this proposed model is relatively high, and this model has not been verified in a large number of clinical trials. In future research work, we will carry out research on the problem of the high computational cost of CBCT image processing by the network at present, adopt more advanced image preprocessing methods to improve the recognition effect, and try the clinical application of other variants of ViT. In addition, we will collect more data in a clinical setting to verify the performance of the model.

## Conclusions

In this paper, a CBCT image classification model based on ViT is proposed, and this model can diagnose the maturation of MPS with high efficiency and accuracy. This paper further explores the ViT model and improves its classification performance by transfer learning and introducing the locality of CNN. The accuracy of the proposed ViT on our test set is 95.75%, which is much higher than the accuracy of the clinician’s judgment. To explain the classification principle of our model, a GradCam-based visual analysis is done for each class of CBCT images. The prediction of the MPS maturity stage given by the ViT proposed in this paper is for the reference of doctors, to greatly improve the work efficiency and diagnostic accuracy of doctors. We believe that this study will be effective in the orthodontic treatment of maxillary expansion.

## Data Availability

The datasets generated and/or analysed during the current study are not publicly available due [To protect patient privacy, the datasets collecting from Guiyang Stomatological Hospital is confidential.], but are available from the corresponding author on reasonable request.

## Abbreviations

MPS: Midpalatal suture
ViT: Vision Transformer
CNN: Convolutional neural network
CBCT: Cone beam computed tomography
RME: Rapid maxillary expansion
SARME: Surgically assisted maxillary expansion
MARPE: Miniscrew assisted rapid palatal expansion
DICOM: Digital Imaging and Communications in Medicine
ROI: Region of interest
NLP: Natural Language Processing
RELU: Rectified Linear Unit
MSA: Multi-Head Self-Attention
MLP: Multi-layer Perceptron
GELU: Gaussian Error Linear Unit
AUC: Area under the receiver operating curve
TP: True Positive
TN: True Negative
FP: False Positive
FN: False Negative
ROC: Receiver operating curve
CAM: Class activation maps
